# Distractor similarity and category variability effects in search

**DOI:** 10.3758/s13414-024-02924-4

**Published:** 2024-07-09

**Authors:** Arryn Robbins, Anatolii Evdokimov

**Affiliations:** Department of Psychology, University of Richmond, 114 UR Drive, Rm 113, Richmond, VA 27303 USA

**Keywords:** Visual search, Categorical search, Category variability, Mental representations, Similarity

## Abstract

Categorical search involves looking for objects based on category information from long-term memory. Previous research has shown that search efficiency in categorical search is influenced by target/distractor similarity and category variability (i.e., heterogeneity). However, the interaction between these factors and their impact on different subprocesses of search remains unclear. This study examined the effects of target/distractor similarity and category variability on processes of categorical search. Using multidimensional scaling, we manipulated target/distractor similarity and measured category variability for target categories that participants searched for. Eye-tracking data were collected to examine attentional guidance and target verification. The results demonstrated that the effect of category variability on response times (RTs) was dependent on the level of target/distractor similarity. Specifically, when distractors were highly similar to target categories, there was a negative relation between RTs and variability, with low variability categories producing longer RTs than higher variability categories. Surprisingly, this trend was only present in the eye-tracking measures of target verification but not attentional guidance. Our results suggest that searchers more effectively guide attention to low-variability categories compared to high-variability categories, regardless of the degree of similarity between targets and distractors. However, low category variability interferes with target match decisions when distractors are highly similar to the category, thus the advantage that low category variability provides to searchers is not equal across processes of search.

## Introduction

In our daily searches for objects or people, we may look for familiar items or faces for which a lot of perceptual detail is available (e.g., your car keys, the face of your child), or for items from categories, where fewer perceptual features of the target are known (categorical search; e.g., look for any trash can). In the laboratory, these types of searches are differentiated by the type of cue presented to the searcher; a picture cue provides substantial perceptual detail for the searcher to utilize for attentional guidance and produces an efficient search, and a word cue requires the searcher to utilize category information from long-term memory. It is understood that the latter categorical search is less efficient than picture-cued search, as the amount of information a searcher has about the appearance of their target is proportional to search efficiency (Malcolm & Henderson, 2009; Schmidt & Zelinsky, 2009).

Despite being less efficient than picture-cued search or search for familiar items, categorical search is still an efficient type of visual search as attention is guided to target items (Schmidt & Zelinsky, 2009; Yang & Zelinsky, 2009). Researchers have been investigating what information is used to guide attention during categorical search, and discovering that category-consistent or category-typical features comprise the guiding mental representation (i.e., the target template) that directs attention to those category features (Hout et al., 2017; Robbins & Hout, 2015, 2020; Yu et al., 2016). Research has also demonstrated that when distractors share features with the target template, as in search scenarios with high target/distractor similarity, there is interference in attentional guidance to targets (Alexander & Zelinsky, 2011, 2012; Duncan & Humphreys, 1989).

Little research has directly examined target/distractor similarity effects in categorical search due to the challenge of manipulating similarity between distractors and real-world images of multidimensional targets or between targets that are categorically defined. However, Alexander and Zelinsky (2011) successfully manipulated target/distractor similarity with category targets, and demonstrated that target/distractor similarity effects extend to categorical search. In their study, human ratings of similarity were obtained between the target categories (teddy bears and butterflies) and a pool of distractor items. The similarity ratings were used to manipulate target/distractor similarity into three conditions: low-similarity distractors, high-similarity distractors, and mixed condition with distractors from low-, medium-, and high-similarity ratings. During target-absent trials, response times (RTs) were shorter when the distractors had low similarity to the target category and fewer distractors were fixated. Additionally, there were more high-similarity distractors fixated during mixed trials, suggesting that attention is guided to shared features between the category representation and distractors, and implying that there is interference in categorical search when there is high similarity between distractors and the category. Other evidence of target/distractor similarity effects is found in studies examining category typicality effects in visual search. If objects in the search environment have some similarity with the target category such as shared features that are typical to the target category, guidance is impaired (Robbins & Hout, 2020) and search efficiency is reduced.

### Category feature variability

In addition to target/distractor similarity, categorical search efficiency is also impacted by the level of precision in the target template. In other words, when a searcher does not have a clear and precise target template for guiding attention, the search becomes more challenging and inefficient (Hout & Goldinger, 2015). This idea is further evidenced by research examining the effects of category variability on search. Category variability (also known as category heterogeneity, consistency, or within-category similarity) refers to the degree to which category members are similar to one another in appearance. In much of the work examining how we search for categories, the target categories are often used equally across key manipulations or are assumed to be comparable to one another. However, more research is uncovering how variability in search performance can be explained by the target categories we search for in daily life and in laboratory tasks. Some categories have members that share consistent features, such as bananas, where category members look very similar to one another and features are easy to predict. Other categories are more variable in appearance, like shoes, and vary in appearance along multiple dimensions like color and shape. The amount of variability a category has in appearance determines the efficiency of search performance (Hout et al., 2017; Yu et al., 2016). Hout et al. (2017) had participants search for two types of stimuli: societally important vehicles that were assumed to have low variability in features (e.g., ambulances, police cars, fire trucks) and civilian vehicles (e.g., cars, trucks, SUVs), which vary more in appearance between exemplars. Search for the low-variability vehicle categories was more efficient as evidenced by better attentional guidance and object recognition compared to the more variable vehicle categories. Additionally, searchers were better able to restrict their attention to target features during search for low-variability categories. The results of Hout et al. (2017) indicated that when searchers are looking for a category with inconsistent and variable features, search is less efficient due to lack of template precision compared to search categories with highly predictable features and low-variability features between category members.

These results, among others (e.g., Hout & Goldinger, 2015), indicate that the amount of information available regarding the appearance of the target is proportionate to search efficiency, with attentional guidance facilitated by the precision of the template. However, this notion that template precision benefits search efficiency is not true in many cases of search. While it may seem like search would be improved by having more perceptual detail available in the template, prior work has suggested otherwise, where template precision can produce inefficient search. In Experiment 2 of Hout et al. (2017), search for low-variability categories was less efficient than high-variability categories when distractors shared features with the targets. In that experiment, participants were provided with a picture cue of the vehicle to search for (e.g., a picture of an ambulance from the side). The search array contained distractors from the same category (e.g., other ambulances from various viewpoints). Attentional guidance and object identification were worse for the low-variability categories (i.e., the societally important vehicles) than the high-variability categories. It may be that the benefits to search efficiency afforded by well-defined targets are only present when distractors are dissimilar from the to-be-sought category.

### Current study

To date, studies examining target/distractor similarity effects on categorical search do not consider the influence of template precision. While we understand the mechanisms that produce target/distractor similarity effects (e.g., attention prioritized to target features that distractors may share), we do not know how the contents of the categorical search template moderate these similarity effects. Recent work by Lavelle et al. (2023) demonstrated that a hybrid search for multiple, related targets (i.e., exemplars from the same category) produced narrow/restricted attention to target features, facilitating search efficiency over search for random/unrelated targets that were variable in their features. Because the related set of targets shared features, searchers were able to capitalize on the consistent features to produce precise templates for efficient guidance of attention. However, when searchers looked for these related targets among novel distractors from the same categories, efficiency was worse compared to search among random, non-category distractors. When searching for the related targets, searchers could efficiently conduct search among random/non-category distractors, but were more likely to confuse same-category distractors as targets and needed additional time to distinguish targets from distractors. So, while participants were utilizing the consistent features to develop precise templates, this produced interference when distractor features overlapped with the template.

We built upon these findings in the domain of categorical search. Rather than having participants search for specific images, our participants were cued with categories of common objects (e.g., butterfly, table, fish). Based on prior research (e.g., Hout et al., 2017; Lavelle et al., 2023), we expected that the degree to which target/distractor similarity affects search depends on the precision of the search template in categorical search. While precise templates help a searcher improve attentional guidance and help distinguish the target from distractors, not every category is equal in terms of providing the searchers with useful information to develop a precise search template. Search for high-variability categories may rely on a coarser template for guidance and verification, as features are less predictable than low variability categories. When searching within environments where there is a high degree of similarity between target and distractors, the coarser high-variability template may provide a benefit over the low-variability template as there may be less interference than with a precise template (e.g., Experiment 2 of Hout et al., 2017).

One of the challenges in examining target/distractor similarity in categorical search is determining the reference from which to manipulate target/distractor similarity. Prior studies examining target/distractor similarity manipulate feature values along single dimensions between target and distractors (e.g., search for a red target among red vs. orange distractors). In the case of categorical search, especially those involving real-world images, it can be challenging to identify and quantify features of targets in order to manipulate the similarity between target and distractors. For example, how can we manipulate the similarity between a categorical target like “dogs” and distractor images in the search array? We used a novel approach of manipulating the similarity between distractor images and category prototypes that relies on multidimensional scaling (MDS; Hout et al., 2016). Using MDS we were able to identify target category-similar and -dissimilar distractors. By using this approach, we were able to manipulate similarity between features of the *best representation* of the category and distractors, not necessarily the similarity between *encountered* target and distractors, as has been done traditionally. Furthermore, this method allowed us to examine the influences of out-of-category similarity between targets and distractors, where other studies produce target/distractor similarity effects by utilizing targets and distractors from the same categories. We also used MDS to quantify category variability of our target categories by conceptualizing category variability as being synonymous with within-category similarity.

## Experiment 1

### Method

Participants searched for categories of objects that had a low degree of within category similarity (i.e., low category variability) or high category variability. The distractors were either highly similar or highly dissimilar to the target category, as determined by MDS distances.

#### Power analysis

To calculate required sample size, we relied on results from Hout et al. (2017), which had a similar design and research questions. Based on this prior work, we anticipated a large effect of variability on search performance and a large effect of target/distractor similarity, as has been established in much of the prior work in this domain. For Experiment 1, we initially conducted an a priori power analysis in GPower v3.1.9.7 (Faul et al., 2009) using the test for ANOVA: repeated-measures, within-factors. We used an effect size of f = .25, a desired power of .80, α = 05, one group with 12 measurements (for the 2 × 2 × 3 design). This suggested a sample size of 13. Because we used linear mixed models (LMMs) for the analysis and for Experiments 2a and 2b, we decided to conduct a power analysis specific to LMM using the *mixedpower* package in R (Kumle et al., 2021; R Core Team, 2023). For this analysis, we conducted a series of simulations to estimate power for given sample sizes (N = 10, 20, 30, or 40), fixed effects and their interactions (variability and target/distractor similarity), and subject as a random variable. The simulations took as input the RT data (correct trials and outliers removed) from Experiment 1 to estimate power for Experiments 2a and 2b (see Tables 1 and 2). Both types of power analyses indicated we were likely well powered (i.e., .8 or above) for all experiments to detect the expected interaction between variability and target/distractor similarity.

**Table 1:** List of target categories used from the PiCS (Pictures by Category and Similarity) database

Category	Average MDS distance	Variability label
**Cabinet**	390.02	Low
**Gun**	396.89	Low
**Table**	426.61	Low
**Teddy bear**	449.46	Low
**Fish**	486.58	Low
**Butterfly**	490.71	Low
**Chair**	547.82	High
**Pillow**	557.19	High
**Clock**	557.95	High
**Hat**	559.85	High
**Car**	573.51	High
**Mug**	575.74	High

*MDS* multidimensional scaling

**Table 2:** Simulated power estimates using fixed effects and varying sample sizes

Effect	Sample size			
	10	20	30	40
**Variability**	.769	.969	.999	.999
**Target/Distractor Similarity**	.932	.999	1.000	1.000
**Variability × Similarity**	.667	.916	.990	.999

#### Participants

Seventeen students from Carthage College participated in the experiment, all of whom were over the age of 18 years. Age and gender information were not collected. Participants were recruited from SONA Systems, the online participant pool. Participants had normal or correct-to-normal vision and all reported normal color vision.

#### Apparatus

The testing room was lit with overhead lighting and floor lamps. The room contained five computers spaced apart from one another on individual desks. Five participants could complete the experiment at a time. The experiment was conducted in EPrime 3.0 (Psychology Software Tools Pittsburgh, PA, USA). On four of the five computers, the stimuli were presented on a 23.6-in. monitor with a refresh rate of 59 Hz and screen resolution of 1,920 × 1,080. On one of the five computers, the stimuli were presented on a 23.6-in. monitor with a refresh rate of 60 Hz and screen resolution of 1,920 × 1,080. Participants were seated approximately 24 in. from the monitor.

#### Design

The design was 2 (Low or High Variability) × 2 (Similar or Dissimilar Distractors) × 3 (6-, 12-, or 18-item set size) within-subjects. There was also a manipulation of target presence (i.e., target present or absent). The primary dependent measure was response time to the target (RT).

#### Stimuli and selection

Similarity between stimuli was manipulated using multidimensional scaling (MDS). MDS is a statistical technique that is used to model the psychological similarity among stimuli (Hout, Goldinger et al., 2013a, Hout, Papesh et al., 2013b, Hout et al., 2016). In essence, MDS transforms similarity data into a reduced-dimensional space while preserving the relative distances between stimuli. A typical MDS analysis that is used in visual cognition research takes in as input human similarity estimates between all items in a stimulus set. These similarity estimates can be obtained in a variety of ways, such as Likert ratings, pairwise ratings, or the spatial arrangement method (SpAM; Hout, Goldinger, et al., 2013a; Richie et al., 2020). A similarity matrix is generated from these estimates. The MDS analysis then uses data-reduction procedures to reduce complexity in the corresponding similarity matrix and outputs a set of coordinates for each item in a hypothetical “psychological space” arranged by *k* dimensions. Using these coordinates, researchers can calculate Euclidean distances between each item and all other items to investigate similarity. The more dissimilar two items are perceived to be, the larger the distance between them in space and across multiple dimensions.

It is important to note that MDS does not require the identification of specific feature dimensions for which to rate similarity. That is, human raters providing similarity estimates are not directed to rate MDS according to particular dimensions such as color, shape, or size. In a sense, the MDS output is agnostic to the dimensions upon which similarity was rated. The technique doesn't impose constraints on the dimensions along which similarities are assessed. Instead, it allows the data to reveal the underlying dimensions or structures that best explain the observed similarities. This agnosticism to specific dimensions offers several advantages. First, MDS can capture similarity judgments based on various dimensions, including those that may not have been explicitly considered by the raters. Second, by representing the data in a lower-dimensional space, MDS simplifies the visualization of complex relationships among items. It can uncover underlying structures that might not be immediately apparent from the raw data. Finally, MDS outputs often provide visual representations that help researchers or analysts gain insights into the underlying relational structures governing the similarity ratings.

##### PiCS database

All stimuli for the current study were selected from the Pictures by Category and Similarity database (PiCS; Robbins et al., 2019). The PiCS database contains 1,200 images across 20 different object categories. Each object has a continuous similarity rating relative to all other objects in the database. Data collection for the PiCS database involved participants providing visual similarity estimates between object images using the spatial arrangement method. Participants were instructed to provide estimates of visual similarity (rather than conceptual similarity) by arranging selected stimuli on a computer screen, with items perceived as being visually similar placed closer together in space on the computer monitor. The similarity data (i.e., average pixel distance between each item and all other items) were then scaled using MDS.

**Fig. 1:**
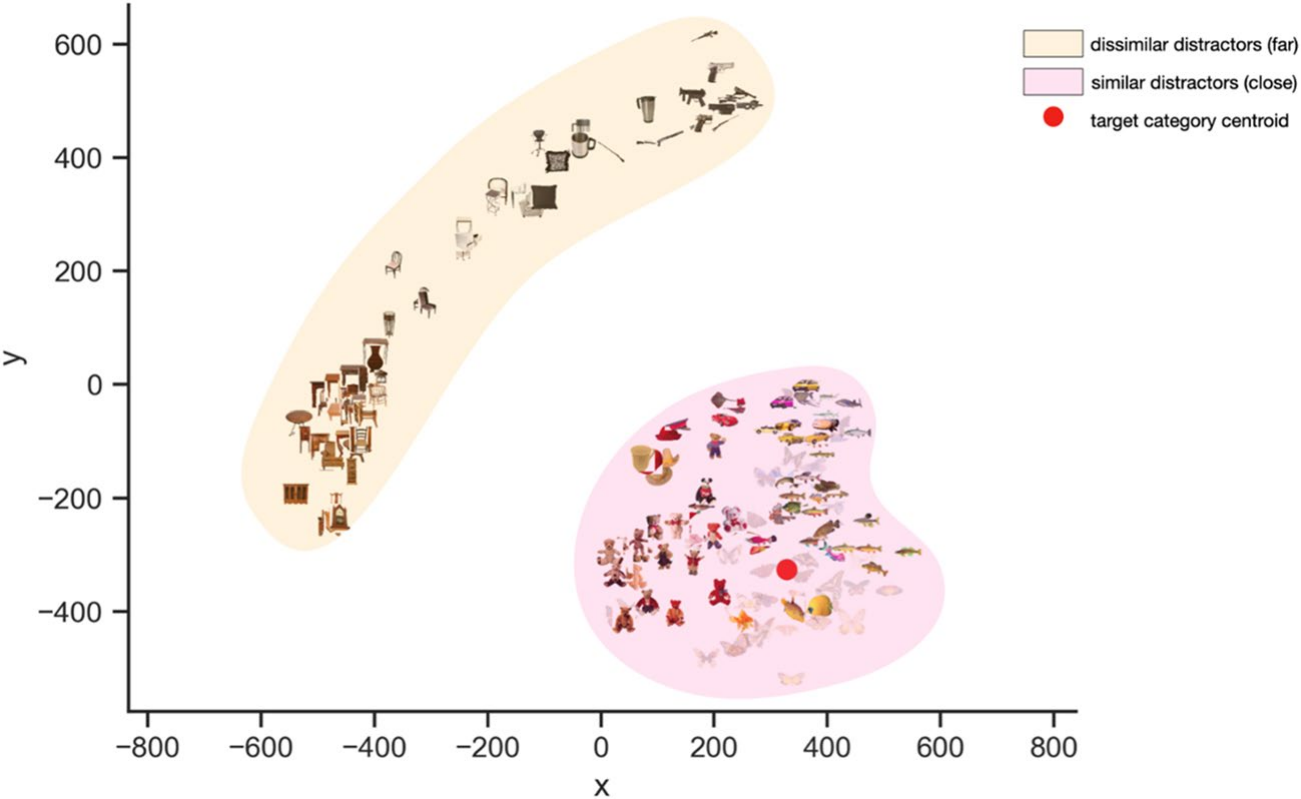
Visual representation of similar and dissimilar distractors for the category “butterfly.” The red dot represents the centroid for the category butterfly. The objects shaded in pink represent the pool of distractors that have the shortest distance to the centroid, and the items in yellow are the furthest from the centroid. The axes represent the first two dimensions output from the MDS analysis

We used the MDS distances between objects in the PiCS database as our measure of similarity, and used these distances to manipulate target/distractor similarity in our trials. For each of the 20 categories in the database, we identified the centroid. This centroid was the object within the multidimensional space that had the shortest average distances to all other objects within the same category (see Fig. 1). Next, for each category, we identified the 70 objects from the PiCS database that had the shortest distances to the category centroid that were not from the same category and 70 objects that had the longest distances from the category centroid. For each trial, distractors were randomly selected from the pool of 70 similar or dissimilar distractors. Objects from target categories could appear as distractors, but these objects never appeared as encountered targets. For example, a particular butterfly could be in the distractor pool for another category (e.g., fish), but would not be the target when participants searched for butterfly.

We also used the MDS distances to manipulate within-category variability of our target categories. For this, we conceptualized variability as within-category similarity. Categories with low variability (bananas) have a high degree of similarity between category members, while high-variability categories (shoes) have less similarity between items within the category. For each category in the PiCS database, we took the average distance between all items within the category. Categories with lower average distances were assumed to have lower variability than categories that had higher average distances (see Table 1 for the categories in order of variability). From the 20 categories, we conducted a tertiary split based on the average within-category distance. we selected six with the lowest averages and six with the highest averages.

#### Procedure

**Fig. 2:**
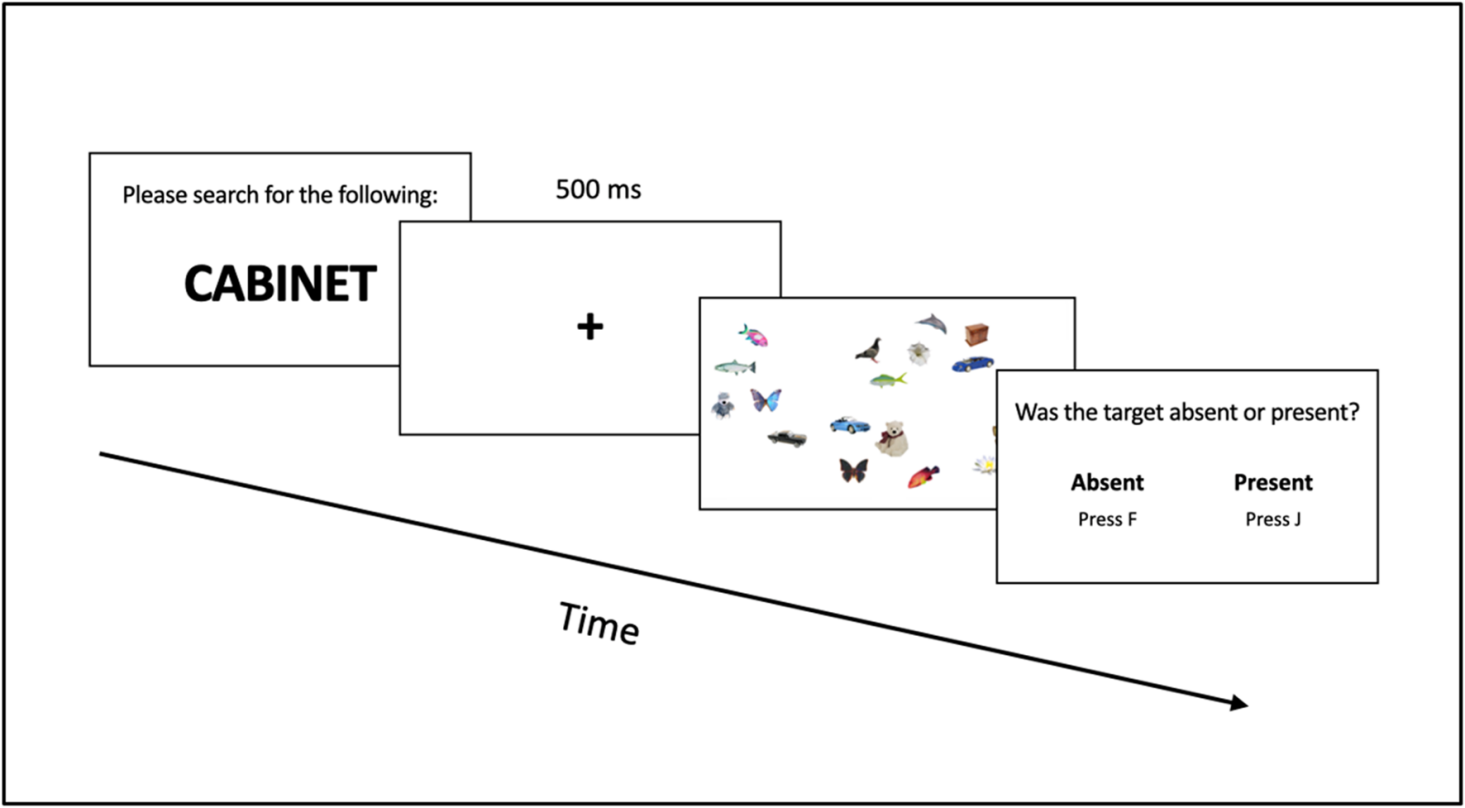
Trial progression for Experiment 1

Participants completed 15 practice trials searching for categories that were not used in the experimental blocks. Following practice, they completed 48 trials in a block, with three blocks for a total number of 144 trials. Fifty percent of the trials had targets absent from the search array and target presence was randomized across trials within each block. During search, participants were told to respond whether the target was present as quickly and accurately as possible. At the start of each trial, a word cue was displayed (see Fig. 2). This cue remained on-screen until the participant was ready to proceed to the search display by pressing the spacebar. Next, the participant saw a fixation cross for 500 ms to center fixation prior to search. The participant then searched for a single target (which was set in a random location on each trial) in an array containing six, 12, or 18 images. The images were resized to 150 pixels on the longest side, with the original propositions maintained. Participants responded whether targets were present (J key) or absent (F key). Targets never appeared at central fixation, requiring at least one eye movement to complete the search.

### Experiment 1: Results and discussion

Overall accuracy was high, 89%, with the lowest accuracy of 79%. No participant data were removed for having an average RT of greater than 3 standard deviations (SDs) above the mean. In both RT and accuracy analyses, although set size was included in the primary analysis, we do not report the set size effects in secondary interactions, rather, we focus on the specific findings of interest, which include a two-way interaction effect between variability and target/distractor similarity and, to examine efficiency, the three-way interaction between variability, similarity, and set size.

#### Response times (RTs)

For all analyses, we performed linear mixed-model (LMM) analysis in The Jamovi (2024) 1.1.9.0 using the GAMLj module (Gallucci, 2019) for LMMs. For our model, we used restricted maximum likelihood for the estimator and included target/distractor similarity, category variability, set size, and their interactions as predictors, as well as a subject-level intercept as a random effect. We converted the variability measure to a continuous measure, rather than use the original dichotomous measure (i.e., low vs. high), by using the average within-category MDS distance for each target category. This approach combined with the use of LMM likely helped improve statistical power over using ANOVA for analysis. The dependent variables included RTs and accuracy. The Satterthwaite method for degrees of freedom was used. Because our primary hypotheses focused on how the effect of category variability differs between similar or dissimilar distractors, we also report the simple effects to see if the effect of variability is present in both distractor similarity types. In the RT analysis, all data are for correct trials only, with an RT cutoff of 4,000 ms. For a summary of parameter estimates from Experiment 1, see Table 3.

**Table 3:** Parameter estimates for the linear mixed models conducted in Experiment 1

Dependent variable	Fixed-effects estimates			Simple-effects estimates		
	Effect	*B*	*SE B*	Trial type	*B*	*SE B*
Accuracy	Intercept	0.888*	0.010	Similar	< .001*	< .001
	Set size	-0.003*	0.001	Dissimilar	-	< .001
	Target/distractor similarity	0.059*	0.012			
	Variability	< .001*	< .001			
	Target/distractor similarity × variability	-*	< .001			
	Variability	-	< .001			
	Set size × target/distractor similarity	0.007*	0.002			
	Set size × target/distractor similarity × variability	< .001	0.002			
	R^2^ for fixed effects	0.02				
Response Time (Target Present)	Intercept	1297.35*	63.33	Similar	-1.87*	0.50
	Set size	30.71*	2.95	Dissimilar	0.44	0.47
	Target/distractor similarity	-385.95*	33.94			
	Variability	-0.72*	0.34			
	Target/distractor similarity × variability	2.31*	0.68			
	Variability	-0.03	0.06			
	Set size × target/distractor similarity	-20.58*	5.91			
	Set size × target/distractor similarity × variability	0.14	0.12			
	R^2^ for fixed effects	0.17				
Response Time (Target Absent)	Intercept	1841.64*	76.20	Similar	-1.26*	0.46
	Set size	64.97*	2.74	Dissimilar	2.37*	0.43
	Target/distractor similarity	-721.69*	31.15			
	Variability	0.56	0.31			
	Target/distractor similarity × variability	3.63*	0.63			
	Variability	-0.01	0.06			
	Set size × target/distractor similarity	-21.45*	5.4s9			
	Set size × target/distractor similarity × variability	0.26*	0.11			
	R^2^ for fixed effects	0.41				

^*^ *p* < .05 - Negligible value

##### Target-present trials

**Fig. 3:**
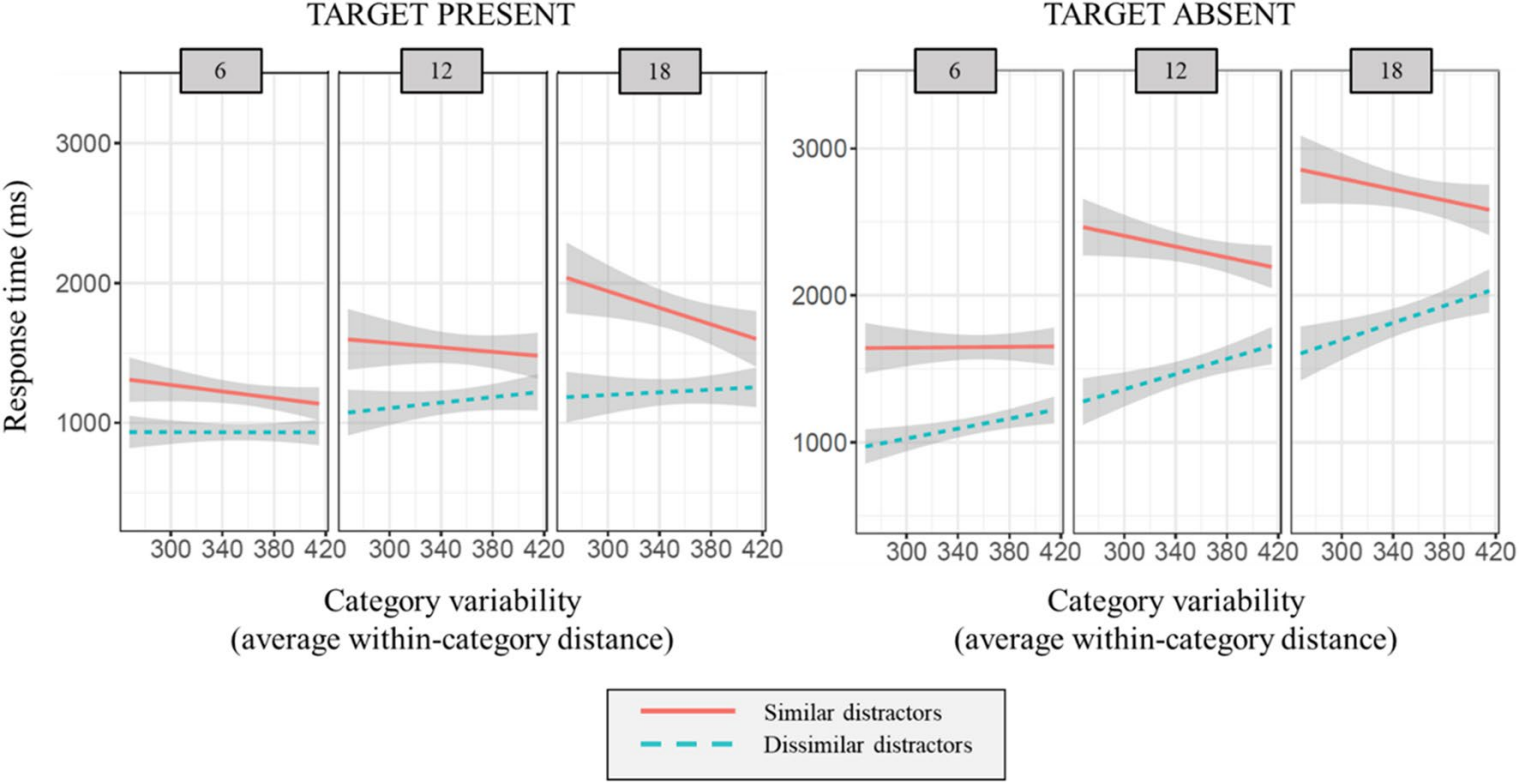
Response times in Experiment 1. Response time here is a function of variability (x-axis), target/distractor similarity (separate lines), and set size (panels). The three panels on the left correspond to trials where the target was present

The mixed-effects model accounted for 31.7% of the variance in RTs, with 16.5% of the variance explained by the fixed effects (i.e., the predictors). There were significant effects of set size (*p* < .001), target/distractor similarity (*p* < .001), and category variability (*p* < .05). Importantly, there was a significant interaction between target/distractor similarity and variability (*p* < .001). Simple effects tests indicated that the effect of variability was present in the trials with similar distractors (*p* < .001), but no effect of variability was present when distractors were dissimilar (*p* = .35). Examination of the slope parameters and the graph indicate that there was a negative relation between variability and RTs, meaning that as the target variability increased, searchers were faster to find the target (see Fig. 3). There was not a significant three-way interaction between variability, target/distractor similarity, and set size (*p* = .23).

##### Target-absent trials

The model accounted for 56.2% of the variance in RTs, with 40.8% of the variance explained by the fixed effects. There were significant effects of set size (*p* < .001), target/distractor similarity (*p* < .001), and no main effect of category variability (*p* = .07). Once again, there was a significant interaction between target/distractor similarity and variability (*p* < .001). Simple effects tests indicated that the effect of variability was present in both target/distractor similarity conditions (*p* < .001), and that the direction of the effect was opposite between the conditions. Specifically, there was a negative relation between variability and RTs in target/distractor similar trials (as was found in the target-present trials), but there was a positive relation between variability and RTs in the dissimilar trials (see Fig. 3). There was a significant three-way interaction between variability, target/distractor similarity, and set size (*p* < .001). Variability was positively associated with RTs for dissimilar distractors (*p* < .001 across all set sizes), and was negatively associated with RTs for similar distractors for set sizes 12 and 18 only (*p* < .01). For set size 6 with similar distractors, there was no significant effect of variability (*p* = .44).

##### Accuracy

**Fig. 4:**
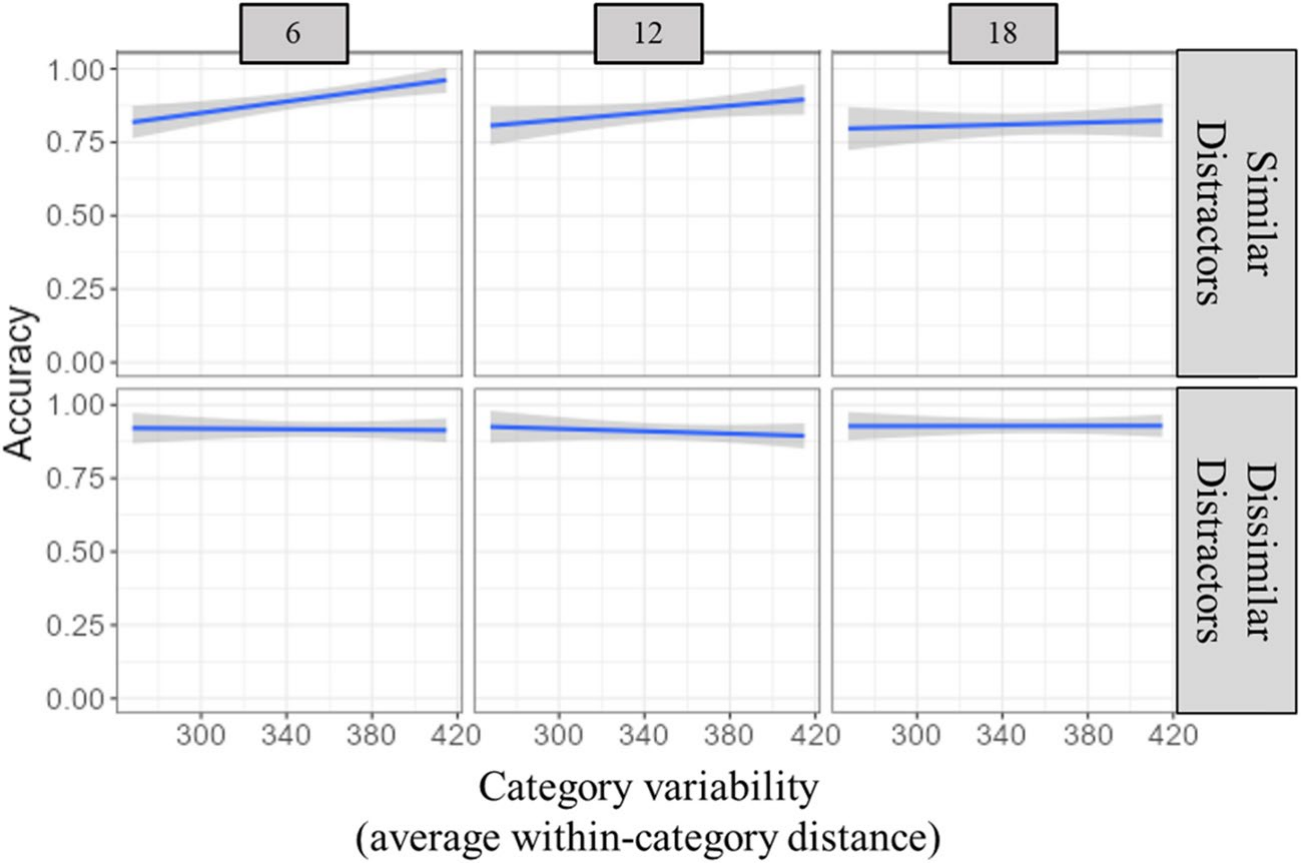
Accuracy in Experiment 1. The average trial accuracy here is a function of variability (X-axis), set size (columns), and target/distractor similarity (rows)

The model accounted for 3.4% of the variance in RTs, with 2.1% of the variance explained by the fixed effects. There were significant effects of set size (*p* < .05), target/distractor similarity (*p* < .001), and category variability (*p* < .05). There was a significant interaction between target/distractor similarity and variability (*p* < .01). Simple effects tests indicated that the effect of variability was present for the target/distractor similar trials only (*p* < .001), and that there was a positive relation between variability and accuracy (see Fig. 4). As variability increased, so did accuracy. There was a no significant three-way interaction between variability, target/distractor similarity, and set size (*p* = .15).

The benefit to searching for low-variability categories that has been found (Hout et al., 2017) goes away when distractors are highly similar or dissimilar to the target category. When searching among similar distractors, decreasing variability of targets was associated with increasing RTs, pointing to a cost to performance that comes with search for low-variability targets. It may also be the case that searching for high-variability targets in these trials was beneficial, due to the broad search template that provides less opportunity for interference from shared target and distractor features. Additionally, the results of Experiment 1 demonstrate that, when items among distractors that are dissimilar, search performance is not influenced by variability as performance was generally the same across variability. This may indicate that our similarity manipulation worked very well, perhaps even creating target pop-out when the distractors were highly dissimilar from the target. However, the finding that variability did not influence RTs in dissimilar trials was only true in the target-present trials. For target-absent trials, and across set sizes, search slopes were steep for dissimilar distractors, with increasing RTs as variability increased.

In Experiment 2 we investigated the individual components of search, including attentional guidance and target verification. This approach allowed us to observe how search templates are shaped by the demands of the task and the search environment. To do this, we used eye tracking, which can precisely record where and for how long a person fixates areas of interest like targets and distractors. Eye tracking was used to examine search templates by observing how participants' visual attention was directed during the search process and how they verified the identity of potential targets in a search array. With this approach we could infer the specificity and flexibility of these search templates, assessing how they adapt based on the nature of the categories being searched for and the similarity of the target to distractors. For example, low-variability targets were expected to produce more efficient attentional guidance than high-variability targets due to template precision, and this would be reflected in faster directing of eye movements to the target. In addition to allowing us to examine how attention is directed to a target, we used eye tracking to see the decision-making processes involved with target/template matching. Once a potential target captures the searcher's attention, they must ascertain whether it indeed matches the search criteria and ultimately their search template in memory. Eye-tracking data, specifically the duration and frequency of fixations on objects, provide critical evidence of this verification process. Longer fixations might indicate more complex verification processes, possibly due to ambiguity in the target's features or a close resemblance to non-target objects within the visual field.

Additionally, in our search task, we expected that color may be the primary driver of the effects on search, especially considering the potential pop-out effect for dissimilar distractor trials. However, MDS is multidimensional, so color is not the only dimension being represented as a dimension upon which humans rate similarity. It may be the case that some of these effects persist when there is no benefit to color. Thus, Experiment 2a utilized the same color stimuli as Experiment 1, and Experiment 2b used the same stimuli but in greyscale.

## Experiment 2

### Method

Participants in Experiment 2 were asked to search for a category while their eye movements were recorded with some changes to the design and procedure compared to Experiment 1. First, the number of stimuli in the search array was kept constant (19 distractors and one target) and targets were always present in the trials. Participants indicated they found the target by pressing the space bar. Then, to check accuracy, participants saw numbers appearing in the place of the stimuli, and they were asked to choose which number corresponded to the target. Stimuli in Experiment 2a were in color and in Experiment 2b were in greyscale, as stated above.

#### Participants

Thirty-one students from the University of Richmond participated in Experiment 2a and 30 participated in Experiment 2b. All were over the age of 18 years. Age and gender information were not collected. Participants were recruited from SONA Systems, the online participant pool. Participants had normal or correct-to-normal vision and all reported normal color vision. One participant was removed for having accuracy below 70%.

#### Apparatus

Participants were tested individually in a dimly lit room. The experiment was conducted in EPrime 3.0 (Psychology Software Tools, 2016, Pittsburgh, PA, USA). The stimuli were presented on a 24-in. LCD monitor with a refresh rate of 60 Hz and screen resolution of 1,920 × 1,080, connected to a computer running Windows 10. Participants sat approximately 40 in. from the monitor. Monocular eye tracking (right eye) was recorded at 500 Hz using an Eyelink 1000+. Participants rested their heads on a chinrest.

#### Design and procedure

**Fig. 5:**
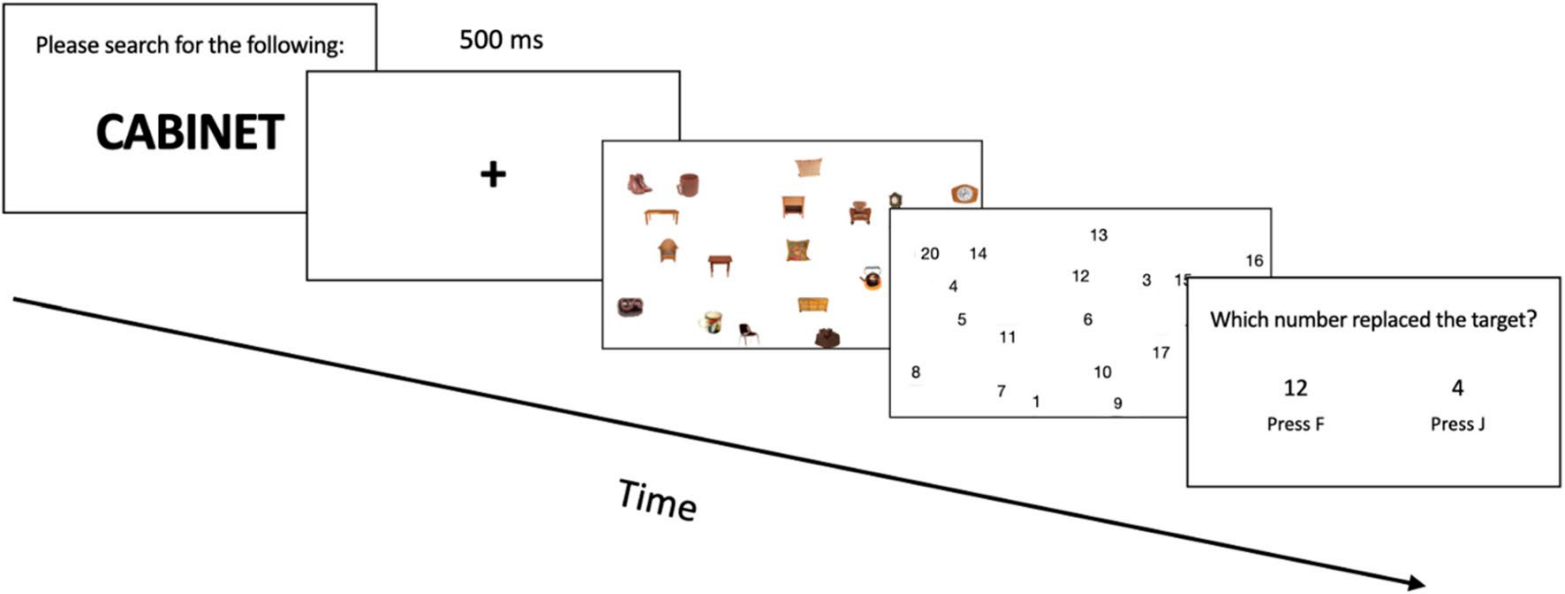
Trial progression for Experiments 2a and 2b

As with Experiment 1, our factors of interest were target/distractor similarity (low and high), and category variability, and both factors were presented within-subjects. For Experiments 2a and 2b, category variability was kept as a continuous measure for analysis. Targets were always present. Participants completed ten practice trials searching for categories that were not used in the experimental blocks. Before beginning the search trials, a nine-point eye-tracking calibration was used to calculate the eye position. During calibration, participants fixated a small black circle that appeared in nine different locations on the monitor. Calibration was accepted if the mean error was less than .5° of visual angle, with no error more than 1° of visual angle. Participants then completed 24 experimental trials in a block, with five blocks for a total number of 120 experimental trials. During the search task, participants were told to respond as quickly and accurately as possible. The trial started with a word cue, followed by a fixation cross, and the 20-item search array. Set size was fixed at 20 with images resized to 100 pixels for the longest side. Participants pressed the spacebar when they located the target. To check accuracy, numbers took the place of the items in the search array and participants had to determine which number took the place of the target with a two-alternative forced choice. This method of search termination allows us to remove the time required for motor action from our search time measure to better reflect termination of the search process (see Fig. 5). Targets never appeared at central fixation, requiring at least one eye movement to complete the search.

### Experiment 2: Results and discussion

The same LMM procedures from Experiment 1 were conducted, without set size as a predictor, and included new analyses for attentional guidance and target verification using eye-tracking data. We conducted analyses separately between Experiment 2a (color stimuli) and 2b (greyscale). Incorrect trials and those where targets were not fixated were not used in the analysis (3.8% of total trials). For each dependent measure, trials that were 3 SDs above the mean were removed, and for time-based measures we used cutoffs for what seemed implausibly short. As with Experiment 1, accuracy was high across conditions (M = 97%, 95% confidence interval (CI) [97.1, 97.8]), and therefore not analyzed further. See Tables 4, 5, 6 and 7 for model parameter estimates.

**Table 4:** Parameter estimates for the linear mixed models conducted in Experiment 2a (color stimuli)

Dependent variable	Fixed-effects estimates			Simple-effects estimates		
	Effect	*B*	*SE B*	Trial type	*B*	*SE B*
Response time	Intercept	1779.75*	135.01	Similar	-1.74*	0.51
	Variability	0.27	0.35	Dissimilar	2.28*	0.48
	target/distractor similarity	-2369.57*	253.25			
	Variability × target/distractor similarity	4.02*	0.71			
	*R*^*2*^ for fixed effects	0.17				
Visits before target	Intercept	2.55*	0.45			
	Variability	0.01*	0.00			
	Target/distractor similarity	-3.92*	0.89			
	Variability × target/distractor similarity	< .01	< .01			
	*R*^*2*^ for fixed effects	0.10				
Time to fixate target	Intercept	1494.44*	120.20	Similar	1.52*	0.48
	Variability	2.03*	0.33	Dissimilar	2.53*	0.45
	Target/distractor similarity	-1107.35*	235.37			
	Variability × target/distractor similarity	1.02	0.66			
	*R*^*2*^ for fixed effects	0.15				
Distractor rejection time	Intercept	248.94*	7.73	Similar	0.02	0.02
	Variability	-0.07*	0.02	Dissimilar	-0.15*	0.03
	Target/distractor similarity	75.90*	13.17			
	Variability × target/distractor similarity	-0.17*	0.04			
	*R*^*2*^ for fixed effects	0.004

**Table 5:** Parameter estimates for the linear mixed models conducted in Experiment 2a (contd.)

Dependent variable	Fixed-effects estimates			Simple-effects estimates		
	Effect	*B*	*SE B*	Trial type	*B*	*SE B*
Run count	Intercept	1.54*	0.08	Similar	< .01*	< .01
	Variability	< .001*	< .01	Dissimilar	< .001	< .01
	Target/distractor similarity	-1.04*	0.15			
	Variability × target/distractor similarity	< .01*	< .01			
	*R*^*2*^ for fixed effects	0.05				
Time to press	Intercept	1443.81*	106.41	Similar	-3.21*	0.41
	Variability	-1.63*	0.28	Dissimilar	-0.05	0.39
	Target/distractor similarity	-1411.55*	201.40			
	Variability × target/distractor similarity	3.16*	0.56			
	*R*^*2*^ for fixed effects	0.04				
Number of fixations after target	Intercept	1.90*	.14	Similar	-.02*	,.01
	Variability	-.01*	0.001	Dissimilar	< .001	< .01
	Target/distractor similarity	-1.57*	.15			
	Variability × target/distractor similarity	.02*	.003			
	*R*^*2*^ for fixed effects	.05				
Target first fixation duration	Intercept	218.97*	3.49			
	Variability	.09*	.04			
	Target/distractor similarity	-14.30*	3.94			
	Variability × target/distractor similarity	.053	.08			
	*R*^*2*^ for fixed effects	.007				

^*^ *p* < .05

**Table 6:** Parameter estimates for the linear mixed models conducted in Experiment 2b (greyscale)

Dependent variable	Fixed-effects estimates			Simple-effects estimates		
	Effect	*B*	*SE B*	Trial type	*B*	*SE B*
Response time	Intercept	1992.18*	146.22	Similar	-0.96*	0.57
	Variability	-0.03	0.39	Dissimilar	1.02*	0.52
	Target/distractor similarity	-1480.41*	276.65			
	Variability × target/distractor similarity	1.98*	0.77			
	*R*^*2*^ for fixed effects	0.11				
Visits before target	Intercept	2.79*	0.46			
	Variability	< .01*	< .01			
	Target/distractor similarity	-3.17*	0.92			
	Variability × target/distractor similarity	< .01	< .01			
	*R*^*2*^ for fixed effects	0.07				
Time to fixate target	Intercept	1583.17*	126.04	Similar	1.30*	0.50
	Variability	1.76*	0.35	Dissimilar	2.21*	0.47
	Target/distractor similarity	-943.74*	248.48			
	Variability × target/distractor similarity	0.90	0.69			
	*R*^*2*^ for fixed effects	0.10				
Distractor rejection time	Intercept	226.47*	7.03	Similar	0.05	0.06
	Variability	0.00	0.02	Dissimilar	-0.57*	0.08
	Target/distractor similarity	30.33*	12.49			
	Variability × target/distractor similarity	-0.06*	0.04			
	*R*^*2*^ for fixed effects	0.002				

^*^ *p* < .05

**Table 7:** Parameter estimates for the linear mixed models conducted in Experiment 2b (contd.)

Dependent variable	Fixed-effects estimates			Simple-effects estimates		
	Effect	*B*	*SE B*	Trial type	*B*	*SE B*
Run count	Intercept	1.73*	0.09	Similar	>-.01*	< .01
	Variability	>-.001*	< .01	Dissimilar	>-.01	< .01
	Target/distractor similarity	-0.57*	0.17			
	Variability × target/distractor similarity	0.001*	< .01			
	*R*^*2*^ for fixed effects	0.02				
Time to press	Intercept	1838.36*	116.33	Similar	-3.35*	0.46
	Variability	-2.41*	0.31	Dissimilar	-1.47	0.43
	Target/distractor similarity	-905.75*	224.86			
	Variability × target/distractor similarity	1,88*	0.63			
	*R*^*2*^ for fixed effects	0.03				
Number of fixations after target	Intercept	2.55*	.17	Similar	-.03*	.002
	Variability	-.01*	0.002	Dissimilar	.002	< .01
	Target/distractor similarity	-1.68*	.11			
	Variability × target/distractor similarity	.02*	.003			
	*R*^*2*^ for fixed effects	.04				
Target first fixation duration	Intercept	226.20*	4.20			
	Variability	.07	.04			
	Target/distractor similarity	-6.74	3.96			
	Variability × target/distractor similarity	.009	.08			
	*R*^*2*^ for fixed effects	.002				

^*^ *p* < .05

#### RT

**Fig. 6:**
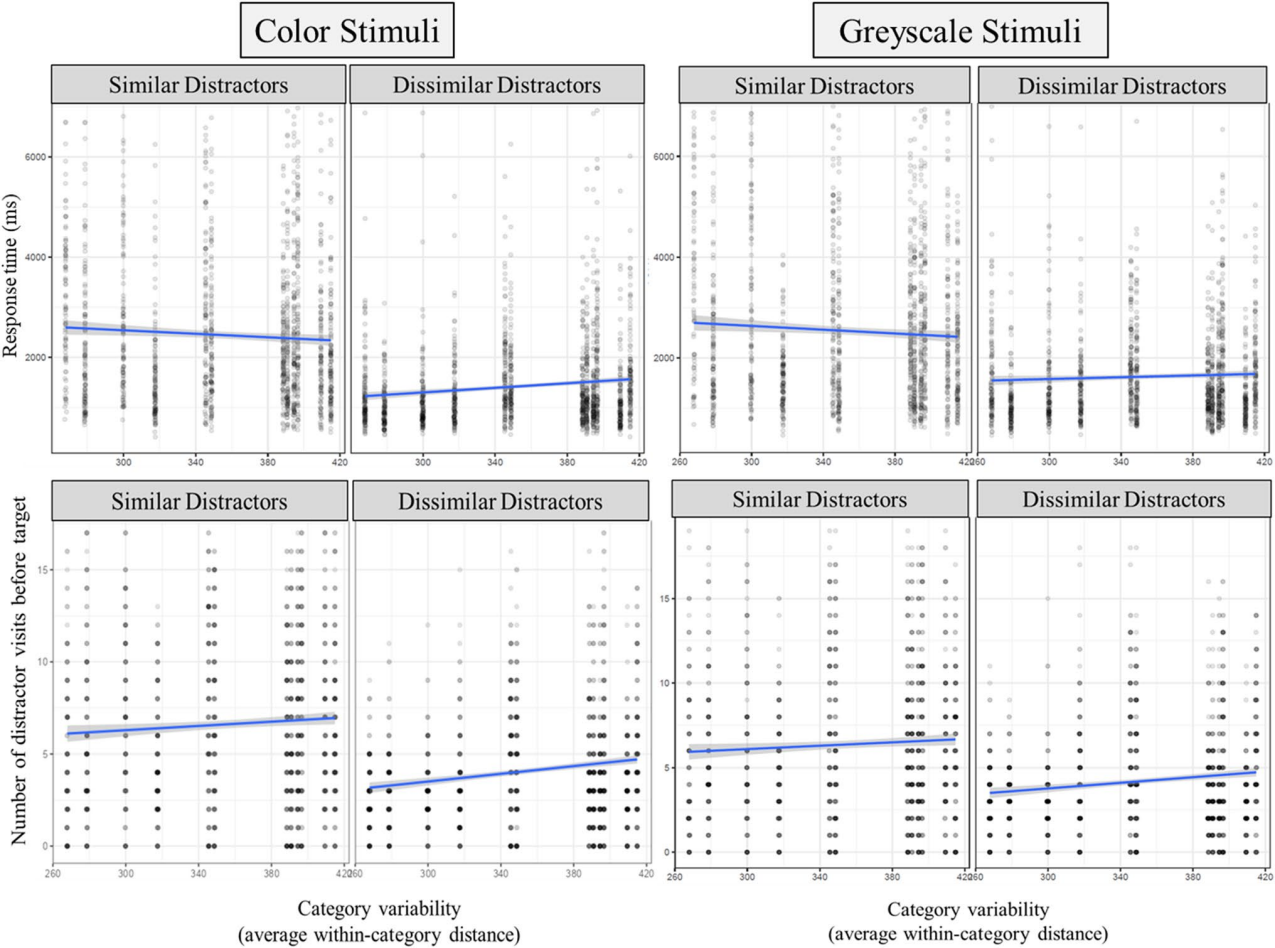
Dependent measures in Experiments 2a and 2b. Response time (top row) and number of distractor visits (bottom row) is a function of variability and target/distractor similarity. The left panels show the results for Experiment 2a, which used color stimuli, and the right panels show the RT results for Experiment 2b, which used greyscale stimuli

The mixed effects model for color stimuli accounted for 22.2% of the variance in RTs, with 17.1% of the variance explained by the fixed effects. There was a significant effect of target/distractor similarity (*p* < .001), no significant effect of category variability (*p* = .49), and a significant interaction between target/distractor similarity and category variability (*p* < .001). Simple effects tests indicated that the effect of variability was present in both target/distractor similarity conditions (*p* < .001). There was a negative relation between variability and RTs in target/distractor similar trials (as was found in Experiment 1), and a positive relation between variability and RTs in the dissimilar trials (see Fig. 6).

For greyscale stimuli, the mixed-effects model accounted for 11.0% of the variance in RTs, with 15.9% of the variance explained by the fixed effects. Trends were similar to the color stimuli, as there was a significant effect of target/distractor similarity, (*p* < .001), no significant effect of category variability (*p* = .93), and a significant interaction between target/distractor similarity and category variability, (*p* < .01). Simple-effects tests indicated there was no effect of variability on RTs in target/distractor similar trials (*p* = .09), and a positive relation between variability and RTs in the dissimilar trials (*p* < .05).

#### Attentional guidance

First, we analyzed whether guidance to targets was significantly better than chance. If attentional guidance to the target was significant, searchers should fixate less than 50% of the set size. We conducted two-tailed, single-sample t-tests to compare the average number of distractor visits before the target to 50% of the set size (i.e., ten items). The number of distractor visits prior to the target was significantly different from ten for both color (*t* = -66.4, *p* < .001, *d* = -1.11 , M_*d*_ = -4.65, 95% CI [-4.65, -4.79]) and greyscale stimuli (*t* = -67.5, *p* < .001, *d* = -1.15 , M_*d*_ = -4.78, 95% CI [-4.88, -4.60]).

Next, we analyzed the number of distractors visited before the target with the LMM model and our established predictors (see Fig. 6). Fewer distractors fixated prior to the target would indicate better attentional guidance. For color stimuli, the mixed-effects model accounted for 10.6% of variance, with 9.6% of the variance explained by the fixed effects. There was a significant effect of target/distractor similarity (*p* < .001), a significant effect of category variability (*p* < .01), and a marginally significant interaction between the predictors (*p* = .06). We report the simple effects to identify potential trends, though they should be evaluated with caution given the marginally significant interaction effect. Variability was a significant predictor in trials with similar distractors (*p* < .01) and dissimilar distractors (*p* < .001). Both slopes were positive, indicating that as variability increased, more distractors were fixated, with a slightly steeper slope in dissimilar trials (*B* = .005 vs. *B* = .01). For greyscale stimuli, the mixed-effects model accounted for 8.0% of variance, with 6.7% of the variance explained by the fixed effects. There was a significant effect of target/distractor similarity (*p* < .001), a significant effect of category variability (*p*< .001), and no significant interaction (*p* = .15).

We also conducted LMM with the time to first fixation of the target (TTF) as the outcome variable. This measure of eye tracking captures scanning time and can include the efficiency of attentional guidance as well as other time-based processes such as the efficiency in rejecting distractors. For the color model, 15.5% of the variance was accounted for by the model, and 13.6% by the fixed effects. There was a significant effect of target/distractor similarity (*p* < .001), significant effect of category variability (*p* < .001), and no significant interaction between target/distractor similarity and category variability (*p* = .20). TTF increased with variability and when the distractors were similar to the target. For greyscale, the model accounted for 11.9% of the variance, with 10.6% explained by the predictors. There was a significant effect of target/distractor similarity (*p* < .001), significant effect of category variability (*p* < .001), and no significant interaction between target/distractor similarity and category variability (*p* = .21). The main effects showed similar trends to the color stimuli, with TTF increasing with variability and when distractors were similar to the target category.

#### Target verification

**Fig. 7:**
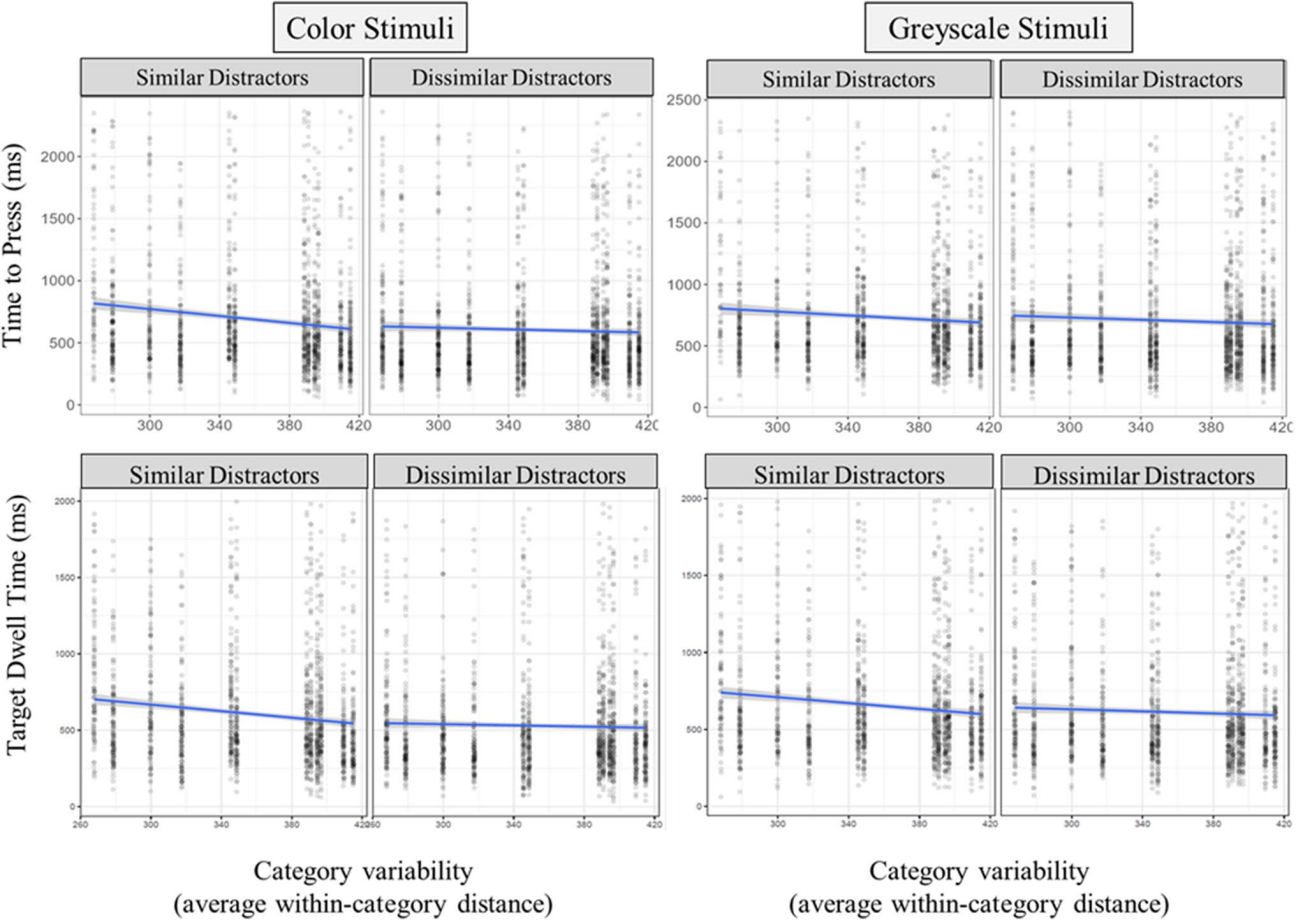
Target verification eye-tracking measures for Experiments 2a and 2b. **Top row:** Time to press, with left two graphs representing color stimuli effects for similar and dissimilar distractor trials, and right graphs representing greyscale stimuli. **Bottom row:** Target dwell time

The process of verifying a target includes the examination of the target once it is fixated and is thought to reflect a target-to-template match decision. This is typically assessed using time-based measures. The time to press (TTP) refers to the time from the first fixation of the target to when participants press the spacebar, and is widely used as a measure of target verification. We used trials in which the target was examined no more than twice. For TTP with color stimuli, the mixed-effects model accounted for 9.3% of the variance, with 4.4% of the variance explained by the fixed effects. There was a significant effect of target/distractor similarity (*p* < .001), significant effect of category variability (*p* < .001), and a significant interaction between target/distractor similarity and category variability (*p* < .001). Simple-effects tests indicated there was a negative relation between variability and TTP in target/distractor similar trials (*p* < .001), and no effect of variability on TTP in dissimilar trials (p = .89). For greyscale stimuli and TTP, the mixed-effects model accounted for 6.5% of the variance, 3.4% explained by the fixed effects. There was a significant effect of target/distractor similarity (*p* < .001), significant effect of category variability (*p* < .001), and a significant interaction between target/distractor similarity and category variability (*p* < .01). Simple-effects tests indicated there was a negative relation between variability and TTP in target/distractor similar trials (*p* < .001), and also in dissimilar trials (*p* < .001; see Fig. 7).

The next measure for target verification is dwell time on the target. The color stimuli model accounted for 10.5% of the variance, with 2.2% of the variance explained by the fixed effects. There was a significant effect of target/distractor similarity (*p* < .001), significant effect of category variability (*p* < .001), and a significant interaction between target/distractor similarity and category variability (*p* < .001). Simple-effects tests indicated there was a negative relation between variability and dwell time in target/distractor similar trials (*p* < .001), and no effect of variability on dwell time in dissimilar trials (*p* = .25). The greyscale model accounted for 8.7% of the variance, with minimal variance explained by the fixed effects (< 1%). There was a significant effect of target/distractor similarity (*p* < .01), significant effect of category variability (*p* < .001), and a marginally significant interaction between target/distractor similarity and category variability (*p* = .05). Simple effects tests indicated there was a negative relation between variability and dwell time in target/distractor similar trials (*p* < .001), and a marginal effect of variability on dwell time in dissimilar trials (*p* = .05; see Fig. 7).

The next measure was the initial target fixation duration. The color stimuli model accounted for 3.1% of the variance, with < 1% of the variance explained by the fixed effects. There was a significant effect of target/distractor similarity (*p* < .001), a significant effect of category variability (*p* < .05), and no significant interaction between target/distractor similarity and category variability (*p* = .49). The greyscale model accounted for 4.1% of the variance, with minimal variance explained by the fixed effects (< 1%). There were no effects of target/distractor similarity (*p* = .09), category variability (*p* = .07), nor an interaction between target/distractor similarity and category variability (*p* = .91).

**Fig. 8:**
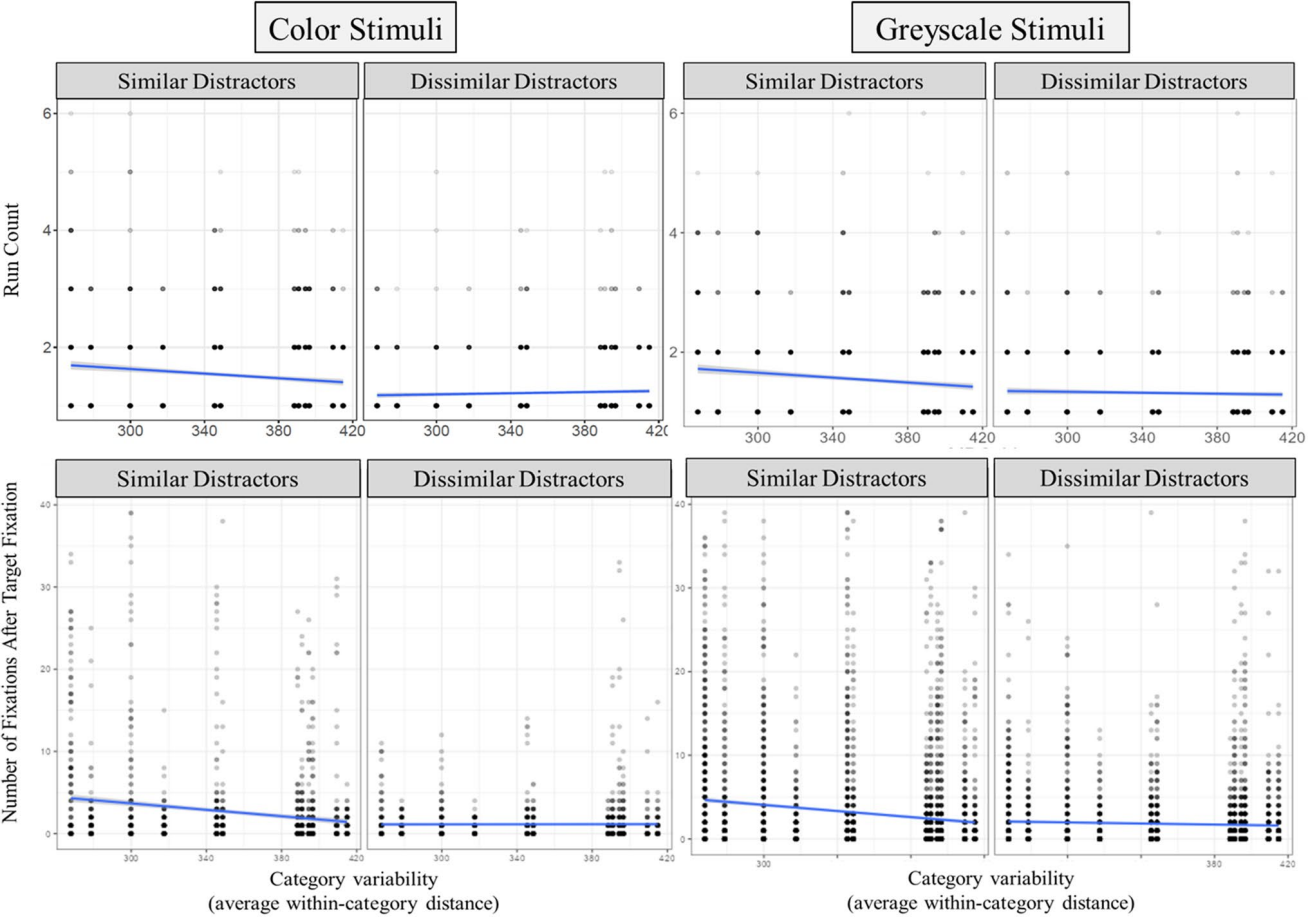
Additional eye-tracking measures for Experiments 2a and 2b. **Top row:** Run count, with left two graphs representing color stimuli effects for similar and dissimilar distractor trials, and right graphs representing greyscale stimuli. **Bottom row:** Number of fixations made after the initial fixation to the target

Target verification may also include comparisons between the target and confusable distractors. If a searcher was not able to develop an adequate template for fast target verification (e.g., due to variability), this may be reflected in multiple examinations of confusable distractors. Thus, we investigated the verification process further by examining how target/distractor similarity and category variability influenced the run count of the target. A run refers to a sequential set of fixations on an interest area. Increasing run counts on a target may indicate increasing confusability between the target and similar looking distractors, as the searcher may have to review distractors to compare with the target. With color stimuli, the mixed-effects model for target runs accounted for 9.1% of the variance, with 4.8% of the variance explained by the fixed effects. There was a significant effect of target/distractor similarity (*p* < .001), significant effect of category variability (*p* < .05), and a significant interaction between target/distractor similarity and category variability (*p* < .001). Simple-effects tests indicated there was a negative relation between variability and run count in target/distractor similar trials (*p* < .001), and no effect of variability in dissimilar trials (*p* = .07). For greyscale stimuli, total model variance accounted for was 3.9%, with 2.3% of the variance explained by the fixed effects. There was a significant effect of target/distractor similarity (*p* < .001), significant effect of category variability (*p* < .05), and a significant interaction between target/distractor similarity and category variability (*p* < .05). Simple-effects tests indicated there was a negative relation between variability and run count in target/distractor similar trials (*p* < .001), and no effect of variability in dissimilar trials (*p* = .22; see Fig. 8).

As with changes in run count, if a searcher were using a strategy of examining confusable distractors, we would expect the number of fixations to be higher for trials where the distractors are highly confusable (e.g., low variability and high similarity). We examined the relationship between our predictors and the number of fixations after the initial fixation of the target. For color stimuli, the mixed effects model accounted for 6.3% of the variance, with 5.3% of the variance explained by the fixed effects. There was a significant effect of target/distractor similarity (*p* < .001), significant effect of category variability (*p* < .001), and a significant interaction between target/distractor similarity and category variability (*p* < .001). Simple-effects tests indicated there was a negative relation between variability and the number of post-target fixations in target/distractor similar trials (*p* < .001), and no effect of variability in dissimilar trials (*p* = .88). For greyscale stimuli, the mixed-effects model accounted for 7.3% of the variance, 4% explained by the fixed effects. There was a significant effect of target/distractor similarity (*p* < .001), significant effect of category variability (*p* < .001), and a significant interaction between target/distractor similarity and category variability (*p* < .001). Simple-effects tests indicated there was a negative relation between variability and the number of post-target fixations in target/distractor similar trials (*p* < .001), and a trending effect of variability in dissimilar trials (*p* = .08; see Fig. 8).

#### Distractor processing

TTF is not a direct measure of attentional guidance; the effect of variability found in TTF may be due to differences in distractor processing and rejection, rather than due to attentional guidance. Because searchers develop a more precise template for low-variability categories, we expected the similar distractors to produce not just interference with guidance to targets, but also in their rejection of distractors. In this case, we would expect longer distractor processing time and slower rejection when viewing distractors that are similar to low-variability targets. To examine distractor processing, we computed the average dwell time per fixation and conducted the same LMM analyses. For color stimuli, the model accounted for 3.4% of the variance, with .5% of the variance explained by the fixed effects. There was a significant effect of target/distractor similarity (*p* < .001), significant effect of category variability (*p* < .001), and a significant interaction between target/distractor similarity and category variability (*p* < .001). Simple-effects tests indicated there was a negative relation between variability and distractor dwell time in target/distractor dissimilar trials (*p* < .001), and no effect of variability on dwell time in similar trials (*p* = .36). Intercepts were lower for target similar trials as well. The greyscale model accounted for 8.7% of the variance, with minimal variance explained by the fixed effects (< 1%). There was a significant effect of target/distractor similarity (*p* < .01), significant effect of category variability (*p* < .001), and a marginally significant interaction between target/distractor similarity and category variability (*p* = .05). Simple-effects tests indicated there was a negative relation between variability and dwell time in target/distractor similar trials (*p* < .001), and a marginal effect of variability on dwell time in dissimilar trials (*p* = .05).

As with Experiment 1, RTs and accuracy had a negative relation with variability for similar distractor trials, indicating that RTs were longer and accuracy was worse for targets lower in variability. When distractors were dissimilar, there was no effect of variability on these measures. The eye-tracking analyses provided some indication as to which processes of search were influenced by the interaction between target/distractor similarity and variability. When examining our measures of attentional guidance (visits) and the time to fixate the target, it is evident that getting attention to the target is facilitated by lower variability, regardless of target/distractor similarity. The eye-tracking measures for attentional guidance pointed to a positive relation between guidance and variability and did not have the interaction effect that was present in RTs and target verification. These results are in line with prior work that suggests the target template contains more useful features for guidance when the target category does not have more variability. Surprisingly, this precision did not produce the expected interference in attentional guidance when distractors were similar to the target category (e.g., experiment 2 of Hout et al., 2017). One explanation is that the categories of stimuli on the low end of our variability measure may not actually be low in variability. Recall that our distinction between low and high variability is relative to all categories in the PiCS database. Our measure of variability should be tested with many more categories to identify stimuli that can provide a wider range of variability scores that may allow for better detection of potential interference. We offer another explanation for this surprising finding in the General discussion that considers the interaction between variability and template resolution differences between the guiding template and the template used during verification.

Eye-tracking measures in Experiments 2a and 2b associated with target verification showed similar trends to RTs, with longer verification times, longer target dwell times, and more target visits and fixations needed for low-variability targets among similar distractors. Variability was not related to these eye-tracking measures when the distractors were dissimilar. When searching among similar distractors, participants likely needed additional evidence to confirm that a fixated object was indeed their target, and this was demonstrated with additional runs and visits to distractors when the target category was lower in variability. So, while guidance was not hindered during search for lower variability categories, target verification was.

The color manipulation had minimal influence on the results. The trends found in RTs and eye tracking were generally the same as the color experiment, except the model intercepts were higher in greyscale for time-based measures, reflecting less efficient guidance and longer verification time. This likely indicates that searchers rely on color for guidance and verification, and forcing participants to prioritize other dimensions for subprocesses of search produced a cost to search performance. Additionally, the interaction effects found in verification measures were trending, and approaching significance, which may indicate that the effects are smaller in grayscale, decreasing our statistical power to detect them. The greyscale results also indicate that the stimuli we used had enough variability in dimensions other than color, as evidenced by the effects of variability detected in the greyscale experiments. This is notable because even though the human similarity ratings used for the PiCS database MDS analysis were not for greyscale stimuli, the database still captures variation in similarity across multiple dimensions, and we were still able to leverage that for our variability manipulation in greyscale.

## General discussion

The purpose of this study was to test whether target/distractor similarity effects in categorical search differ across levels of category variability. We used multidimensional scaling to manipulate target/distractor similarity and to measure category variability. Previous research has indicated that low-variability categories are associated with more efficient guidance and faster target verification compared to high-variability categories (Hout et al., 2017; Yu et al., 2016), likely due to template precision produced by more predictable target features. Building upon prior work examining the effects of category variability on search, results from our experiments show that the effect of variability is not equal across search scenarios, and that search for low-variability categories is hindered when there is a high degree of target/distractor similarity. For trials where distractors were similar, the effect of variability on RTs was inverted, with low-variability categories producing the longest RTs.

We investigated subprocesses of search using eye tracking in Experiment 2 and discovered that the interaction effect between target/distractor similarity and variability in RTs was also present in target verification, but not attentional guidance. Guidance, as measured by the number of distractor visits before the target and the time it took to fixate the target, was influenced by category variability and distractor similarity. Specifically, there was better guidance to targets for lower variability targets and for trials where the distractors were dissimilar from the target category. As mentioned in the discussion for Experiment 2, the lack of the interaction effects in guidance was unexpected, given that prior work in this domain has demonstrated that searchers likely have template precision for low-variability categories and that this precision could produce a cost to guidance when there are similar distractors (Hout et al., 2017).

The second process of search that was investigated was target verification. The time to verify the target and total dwell time on the target both demonstrated a similar trend to RTs, in that categories with lower variability produced the longest dwell times and time to press when distractors were similar to the target. These results may indicate that searchers are challenged by the high degree of similarity between the category features in their target template and the distractor features. The extra time required to confirm low-variability targets could stem from the longer time needed to match the target with its template during target fixation, repeated checks of similar distractors, or a combination of both factors. The results from the initial target fixation duration suggest that target/distractor similarity and variability each independently influenced the time initially spent examining the target; however, the interaction effect found in the other verification measures was not present. Searchers spent less time initially fixating on targets with low variability and low distractor similarity. To then explain the interaction effects found in the other time-based measures of target verification, we considered that searchers may have needed to make additional examinations of highly similar distractors when searching for low-variability targets. We examined target run counts and post-target fixations, which would both indicate if searchers made additional visits to distractors after first visiting the target. We found that searchers made more target runs and post-target fixations when searching for lower variability targets when the distractors were similar. For searches among dissimilar distractors, variability did not influence the measures; searchers neither increased nor decreased their examination of distractors, likely finding it easy to identify targets due to the high dissimilarity, regardless of variability. Together, these eye-tracking findings indicate that searchers either failed to recognize a target upon initially fixating it or needed additional evidence to make a target-match decision by re-examining distractors.

### Distinct influences on subprocesses of search

Our results extend current models of search by demonstrating that category variability and target/distractor similarity exert distinct influences on attentional guidance and target-verification processes during search for categories. Namely, the results from the eye-tracking measures differ between processes of guidance and verification. This leads to the question of how can low variability be optimal for attentional guidance, regardless of distractor context, yet a hindrance to processes of target verification. Prior work examining search templates in categorical search assumed that one template is used for processes of attentional guidance and verification, and much work has found that manipulations that affect attentional guidance also affect verification (e.g., Hout et al., 2017; Robbins & Hout, 2015, 2020). Recent work has shown that the template used during guidance is “fuzzy” compared to the template used for target match decisions. That is, the template for guidance is a coarser representation than that used during verification (Yu et al., 2022). This may indicate two separate representations are used during search: one coarse, guiding template maintained in working memory and target-match decisions require a more detailed and feature-specific template from long-term memory (Wolfe, 2021; Yu et al., 2022, Yu, Zhou, et al., 2023b). Regardless of the mechanisms driving differences in template resolution during guidance and verification, it may be that any cost to performance due to low variability is happening as a result of the higher detail in the template used during verification rather than during guidance. It appears that the difference in information maintained in the guiding template between low- and high-variability categories is large enough to influence search, despite the fuzzy resolution. The precision of a low-variability template, while fuzzy, is still useful in directing attention more efficiently than higher variability categories. However, when making target-match decisions for low-variability targets, relying on a more detailed template for target match decisions seems to open the door for interference and confusability with similar distractors.

What are the possible mechanisms behind such a result? One possibility is that searchers are optimally tuning the guiding template to distinguish it from similar distractors (Geng et al., 2017; Hamblin-Frohman & Becker, 2021; Yu, Rahim et al., 2023a) by placing greater attentional weight on one dimension over others (Lee & Geng, 2020; Müller et al., 1995). Once searchers learn the distractor context they are searching in, templates may shift from weighing diagnostic color features more heavily to weighing another, more reliable dimension like shape to facilitate search (Lerebourg et al., 2023). It may be that our low-variability categories had less variation in features across multiple dimensions other than color compared to high-variability categories, and searchers could still guide attention more effectively without color information. However, once objects are fixated, the target match decision relies on a less flexible template from LTM, one that perhaps includes color in greater resolution (or another diagnostic dimension), and this is where confusion can happen.

Finally, we consider that target verification during high target/distractor similarity searches may have occurred by comparing an attended item to distractors, rather than the attended item being compared immediately to a template in memory. Under this view, perhaps a target template memory is not detailed enough to make a template-match decision upon initial fixation, thus searchers assume the strategy of comparing distractors to a selected item and this would be reflected in additional target runs and fixations post-target fixation. Difficulties in target verification would be based on difficulties in discriminating low-variability items from highlight similar distractors through direct comparison of items in the array, rather than a template in memory. Of course, this line of reasoning is speculative and should be followed up formally with testing.

### Future directions

It is worth pointing out the limitations of the stimuli database that was used so that future work can more powerfully investigate categorical search. First, the similarity ratings used to develop the PiCS database were in color, not greyscale. While we did find similar effects between color and greyscale in our study, it may be that manipulations of target/distractor similarity and category variability using MDS are different when using a featural space derived from ratings in greyscale. Secondly, as mentioned previously, the categories used from the PiCS database may not provide a wide range of variability scores that reflect the potential range of variability estimates in natural categories of stimuli. Future investigations in this domain could identify categories that are low in variability and use the same approach with multidimensional scaling to acquire a measure of the category variability.

Another consideration for future work is the influence of task demands and attentional tuning during search for categories. A primary assumption of the current study and prior work in this domain is that searchers used a static category representation, perhaps developed from experience and category learning, to guide attention and make target match decisions. This also includes the assumption that the representation reflects the variability of the exemplars in the category as they naturally occur. However, because target categories repeated in the current study, it is plausible that searchers learned the featural space of each category as trials progress. Much work has demonstrated that search is highly sensitive to statistical regularities, including the extent to which attention is biased to featural regularities of simple and categorical targets (Addleman et al., 2022; Bahle et al., 2021; Sha et al., 2017). While prior work has demonstrated that long-term memory and category representations can be used to direct attention (Carlisle et al., 2011; Giammarco et al., 2016), and it is not unreasonable to consider that searchers adapt their templates to the featural variation of the target categories during the task (Witkowski & Geng, 2022) rather than utilize a static representation that reflects their prior experience with the category (e.g., a representation from long-term memory). Future research should examine how searchers’ representations of target category feature spaces change during learning and online during search, and further understand the relationship between working memory and long-term memory representations used during categorical search.

## Conclusions

In two experiments we used a novel approach to manipulating target/distractor similarity of categories using MDS and a novel way of measuring variability with MDS. We found that the greater efficiency in search provided by low-variability categories was eliminated when the distractors were similar to the target category. Importantly, our results point to a dissociation of these effects between processes of search. Namely, that low category variability produced more efficient guidance, regardless of distractor similarity. Searchers were better able to select low-variability targets. However, once selected, there was a hindrance to target verification when distractors were similar to the target category.

## Data Availability

Data from all experiments are openly available via the Open Science Framework at https://osf.io/3bg9w/

## Code availability

Code for experimental programs and data analyses are available upon request.
